# Assessing the co-occurrence of intimate partner violence domains across the life-course: relating typologies to mental health

**DOI:** 10.3402/ejpt.v5.24620

**Published:** 2014-09-12

**Authors:** Cherie Armour, Emma Sleath

**Affiliations:** 1 School of Psychology, University of Ulster, Coleraine, UK; 2 Department of Psychology and Behavioural Sciences, Coventry University, Coventry, UK

**Keywords:** Intimate partner violence, inter-generational transmission of violence, polyvictimisation, psychiatric morbidity, anger, alcohol

## Abstract

**Background:**

The inter-generational transmission of violence (ITV) hypothesis and polyvictimisation have been studied extensively. The extant evidence suggests that individuals from violent families are at increased risk of subsequent intimate partner violence (IPV) and that a proportion of individuals experience victimisation across multiple rather than single IPV domains. Both ITV and polyvictimisation are shown to increase the risk of psychiatric morbidity, alcohol use, and anger expression.

**Objective:**

The current study aimed to 1) ascertain if underlying typologies of victimisation across the life-course and over multiple victimisation domains were present and 2) ascertain if groupings differed on mean scores of posttraumatic stress disorder (PTSD), depression, alcohol use, and anger expression.

**Method:**

University students (*N*=318) were queried in relation to victimisation experiences and psychological well-being. Responses across multiple domains of IPV spanning the life-course were used in a latent profile analysis. ANOVA was subsequently used to determine if profiles differed in their mean scores on PTSD, depression, alcohol use, and anger expression.

**Results:**

Three distinct profiles were identified; one of which comprised individuals who experienced “life-course polyvictimisation,” another showing individuals who experienced “witnessing parental victimisation,” and one which experienced “psychological victimisation only.” Life-course polyvictims scored the highest across most assessed measures.

**Conclusion:**

Witnessing severe physical aggression and injury in parental relationships as a child has an interesting impact on the ITV into adolescence and adulthood. Life-course polyvictims are shown to experience increased levels of psychiatric morbidity and issues with alcohol misuse and anger expression.

A recent systematic review estimated that intimate partner violence (IPV) prevalence is 38% in family medicine and 40% in emergency medicine (Sprague et al., 2014). IPV is therefore a pervasive societal problem, one that results in significant aversive social and health outcomes including both physical and psychological illness (Carmichael, 2008). To date, research has highlighted that witnessing IPV in parental relationships has significant long-term consequences for children including the risk of IPV involvement in their own later romantic relationships (Carmichael, 2008; Ehrensaft et al., 2003; Manning, 2011). Exposure to inter-parental conflict in the childhood home increases the risk for IPV in later romantic relationships (Cannon, Bonomi, Anderson, & Rivara, 2009; Levendosky, Huth-Bocks, & Semel, 2002). Furthermore, experiences of IPV in adolescence have been acknowledged as a significant precursor to IPV in both young adulthood (Smith, White, & Holland, 2003) and later adulthood (O'Leary, Malone, & Tyree, 1994). The inter-generational transmission of violence (ITV) hypothesis (Egeland, 1993; Kerley, Xu, Sirisunyaluck, & Alley, 2010) proposes that children exposed to inter-parental violence carry violence into their later relationships, in turn exposing their own children to violence, perpetuating a cycle of violence.

ITV theory proposes that children exposed to violence in their families of origin learn that violence is an appropriate and acceptable approach for resolving interpersonal conflicts (Egeland, 1993). ITV theory is rooted within Bandura's Social Learning Theory (SLT: Bandura, 1973, 1977). The basic premise of SLT in relation to ITV is that children learn how to behave by witnessing IPV within their parents’ romantic relationships, and then through modelling, use this learned behaviour in their own future romantic relationships. This proposal has been widely supported in the empirical literature (Kerley et al., 2010; Renner & Slack, 2006). Indeed, a number of studies have reported that both witnessing and directly experiencing violence in the family of origin increases the likelihood that an individual will both perpetrate and/or be victimised from IPV in later romantic relationships (e.g., Ehrensaft et al., 2003). However, the association between witnessing IPV and later victimisation may differ by gender, with significant links between witnessing IPV and later IPV victimisation found for females but not for males (Temple, Shorey, Tortolerno, Wolfe, & Stuart, 2013; Stith et al., 2000).

Victimisation can occur in many forms, for example, psychological abuse, physical abuse, and sexual abuse. However, studies frequently choose to focus upon one particular type of abuse (Higgins & McCabe, 2001), despite it being apparent that a significant proportion of individuals are victimised across multiple domains (Ford, Grasso, Hawke, & Chapman, 2013; Nooner et al., 2010; Pears, Kim, & Fisher, 2000). Limiting research to examining only one type of abuse does not allow researchers to investigate outcomes specific to multiple abuse history typologies (Turner, Finkelhor, & Ormrod, 2010). Recognition of this point has led to a separate body of research that has focused upon the polyvictimisation of violent events. This research has examined the component parts of victimisation, for example, physical abuse or sexual abuse, and has assessed whether individuals are typically abused across one or more domains.

In investigating polyvictimisation, many researchers have used the statistical methods of latent class analysis and latent profile analysis (LCA and LPA; Hagenaars & McCutcheon, 2002). These methods are ideal for investigating typological hypotheses as they rely on participants responding across a number of indicators; as such, these have been termed person-centred approaches. LPA and LCA are statistical methods which create groupings of individuals based on their similarity of responding to a number of indicators. Translating this to research on IPV, individuals can be grouped together based on the types of IPV experiences they report, thus, enabling the empirical investigation of polyvictimisation. Recent literature examining experiences of victimisation, for example, child maltreatment, have demonstrated clear evidence for the occurrence of polyvictimisation using these methods, demonstrating the usefulness of this technique (Armour, Elklit, & Christoffereson, 2014; Cavanaugh, Martins, Petras, & Campbell, 2013; Ford et al., 2013).

Research pertaining to both the ITV hypothesis and polyvictimisation has been concerned with the psychological outcomes for victims. Pico-Alfonso et al. (2006) reported that women who were physically or both physically and psychologically abused by their romantic partners had a higher severity and incidence of psychiatric morbidity in the form of depression, anxiety, posttraumatic stress disorder (PTSD), and suicidal thoughts compared to non-abused women. Moreover, severe depressive symptoms were noted by women who additionally reported sexual violence. A consistent finding is that the cumulative effects of multiple trauma exposures confer greater risk for severe forms of psychiatric symptomatology (Shevlin, Houston, Dorahy, & Adamson, 2008). Cavanaugh et al. (2013) and Ford et al. (2013) both reported that polyvictimisation confers greater risk for psychiatric morbidity and psychological risk. It is therefore logical that life-course polyvictimisation in IPV, whereby individuals are exposed to various forms of IPV, at multiple points in their life, are likely to report the greatest degree of psychiatric morbidity.

To date, no study has attempted to join these two bodies of literature to assess if polyvictimisation of IPV occurs across the life-course, thus supporting the ITV hypothesis and the polyvictimisation hypothesis simultaneously. Furthermore, no research has examined how such typologies may differ on factors such as psychiatric morbidities.

## Current study aims

LPA was used to ascertain if underlying typologies of victimisation over the life-course and over multiple victimisation domains were present in a sample of students. To achieve this aim, students were surveyed cross-sectionally and asked to retrospectively recall 1) victimisation experiences of witnessing IPV of a parent from a parent's romantic partner, 2) the direct exposure to IPV in their own adolescent romantic relationships (13–17 years), and 3) in their own adult romantic relationships (18+ years). Victimisation was queried in relation to psychological aggression (PA), physical assault (PAS), injury (I), and sexual coercion (SC). The second aim of the study was to identify if there were mean differences on scores between latent groupings based on a number of known psychological correlates of IPV— PTSD, depression, anger expression, and alcohol use.

These aims resulted in two hypotheses. First, that underlying typologies of victimisation would exist. Given the novelty of this study, we did not hypothesise about the nature or number of resultant typologies, with one exception—that a typology of polyvictimisation across the life-course would be found. Second, that the correlates of PTSD, depression, anger expression, and alcohol use would evidence the highest mean scores within the typology that reported the greatest degree of polyvictimisation over the life-course.

## Methods

### Procedure/participants

The data presented here was collected from Northern Irish university students. All participants were approached on campus. Participants were included in the current study if they were university students aged 18 or over, who reported experiencing at least one intimate partner relationship. All were fully informed about the study and all provided written consent prior to completing the questionnaire. Anonymity was ensured given the questionnaire did not collect any identifying information. Completed questionnaire were returned via a locked box. Only the lead author had access to the completed questionnaires. The study was approved by a university ethics committee and encompassed a total of 318 participants.

### Measures

#### The Conflict Tactics Scale

The Conflict Tactics Scale (CTS-2) is a widely used measure of violence within intimate relationships, which measures both perpetration and victimisation by asking behaviourally specific questions across a number of subscales (Straus, Hamby, Buncy-McCoy, & Sugarman, 1996). In total, the CTS2 contains 78 items (39 perpetration and 39 victimisation) spanning five subscales: PA, 8 items; PAS, 12 items; injury (6 items; SC, 7 items; and N, 6 items. Within the current study, all items had two response options (e.g., 0=No; 1=Yes). Three versions of the CTS were used: 1) respondents’ knowledge of the events occurring in their parents’ romantic relationships, 2) events occurring in the participants’ adolescent (13–17) romantic relationships, and 3) events occurring in the participants’ adult (18+) romantic relationships, thus constituting a parent, adolescent, and adult measure, respectively. In total, 11 CTS subscales were created; three PA, three PAS, three injury subscales, and two SC subscales, representing the parent, adolescent, and adult measures (the subscale of SC was not used in the parent questionnaire as recommended by Straus, 2001). All subscales were created by summing items within the relevant domain to create count variables that were used within further analyses. Straus (2007) reported that an assessment of 41 articles using the CTS-2 has deemed the measure reliable. The internal consistency of the CTS items pertaining to victimisation in the Parent (P), Adolescent (AD), and Adult (A) measures of the CTS in the current study were high (Cronbach's alpha coefficient=[P] 0.86; [AD] 0.85; [A] 0.84). The individual subscales used herein, across the three CTS measures, also displayed high internal consistency ranging from 0.63 to 0.83, with the exception of the injury subscales for adolescent and adult reports ([AD] 0.15; [A] 0.24). Note that low reliability estimates of subscales are likely a function of the low prevalence of positive endorsement of these items in any given sample (Straus, 2007).

#### PTSD symptom scale-self-report

The PTSD symptom scale-self-report (PSS-SR) is a 17-item self-report measure assessing the 17 DSM-IV PTSD symptoms (Foa, Riggs, Dancu, & Rothbaum, 1993). In line with changes outlined by the DSM-5, the measure was amended to reflect changes in PTSD criteria; in doing so, additional symptoms (three items) have been added and some existing symptoms have been revised. Participants answered each item based on a 4-point Likert-type scale (0=not at all to 3–5 times or more per week/very much/almost always). The modified PSS-SR has been shown to have high internal consistency (Contractor et al., 2014 [*α*=0.96]). The internal consistency of the PSS-SR in the current study was high (*α*=0.96).

#### The Patient Health Questionnaire

The Patient Health Questionnaire (PHQ-9; Spitzer et al., 1994) measures the symptoms of depression as outlined in the DSM-IV. Typically, participants are queried about symptoms occurring within the previous 2 weeks; however, to be consistent with the PSS-SR, symptoms of depression were queried as they occurred in the previous 4 weeks. Each item comprises four response options ranging from 0=not at all to 3=nearly every day. Scores are summed as a measure of severity and range from 0 to 27. Internal consistency has previously been reported as ranging from 0.86 to 0.89 (Kroenke, Spitzer, & Williams, 2001). The internal consistency of the PHQ-9 in the current study was high (*α*=0.89).

#### Alcohol Use Disorders Identification Test

The Alcohol Use Disorders Identification Test (AUDIT) is a 10-item self-report measure of alcohol usage with three domains—hazardous alcohol usage, dependence, and harm (Saunders, Aasland, Babor, De le Fuente, & Grant, 1993). Scores are summated and range from 0 to 40. Scores above eight indicate hazardous levels of alcohol usage and scores above 20 indicate that alcohol dependence may be an issue and so further diagnostic evaluation is recommended. The AUDIT has been shown to be both valid and reliable across a diverse range of samples (e.g., Reinhart & Allen, 2002). The internal consistency of the AUDIT within the current study was high (*α*=0.81).

#### Dimensions of Anger Reaction

The Dimensions of Anger Reaction (DAR-7) is a self-report measure of anger reaction consisting of seven items (Novaco, 1975a). Typically, the scale items consist of nine response options ranging from 0=not at all to 8=exactly so. Scores are summed and result in a range of scores from 0 to 56. However, a recent study assessing the psychometric properties of the DAR-7 concluded that the degree of anger could be measured by using five response options just as effectively as by using nine response options (Forbes et al., 2004). Therefore, the five response options were used across all seven items within the current study. These ranged from 1=none of the time to 5=all of the time. Items are summed to create severity scores, ranging from 7 to 49, with higher scores equating to higher anger levels. In line with the other measures used, participants were queried about anger as it pertained to the past 4 weeks. The internal consistency of the DAR-7 within the current study was high (*α*=0.83).

### Data analysis

Prior to conducting the analysis, the participants who did not report experiencing a romantic relationship between the ages of 13 and 17 or were missing a response for this question were excluded. LPA was conducted using Mplus 6.12 software (Muthén & Muthén, 1998–2010). LPA is based on continuous indicators and uncovers homogeneous groupings from within a heterogeneous overarching sample. The 11 subscales of the CTS, as noted, were used as indicators in the LPA. Given that the subscale indicators represented count variables, and are substantially skewed with a Poisson distribution, all indicators of the latent class model were identified as “counts” within the Mplus syntax. This method was preferred over recoding the indicators to categorical items, given that retaining the count variables retains more information and thus provides a more informative class structure.

LPA models of increasing size were specified and estimated until reaching a point whereby additional classes were no longer necessary (i.e., non-convergence of models or extremely low class prevalences). All models were estimated using Mplus's default robust maximum likelihood (MLR) estimator. In line with standard practice and published guidelines, LPA models were evaluated and compared across a number of statistical fit indices—Akaike Information Criteria (AIC; Akaike, 1987), Bayesian Information Criterion (BIC; Schwartz, 1978), the sample size adjusted BIC (SSABIC; Sclove, 1987), the bootstrapped Lo-Mendell–Rubin adjusted likelihood ratio test (BSLRT; Lo, Mendell, & Rubin, 2001), and the entropy statistic (Ramaswamy, DeSarbo, Reibstein, & Robinson, 1993). Lower values of the AIC, BIC, and SSABIC indicate more optimal fitting models. A non-significant BSLRT value (*p*<0.05) for a particular LPA model suggests that little value is added by the model with one additional class compared to the model with one less class. Entropy measures classification, with values approaching unity indicating clearer classification. Subsequent to model selection, class comparisons were made in relation to mean PTSD, depression, alcohol use, and anger expression scores using one-way ANOVAs.

## Results

The study included a total of 318 participants. The majority of participants were female (*n*=260; 81.8%), ranged in age from 18 to 48 (*M*=22.56, SD=6.14), and were Caucasian (*n*=315; 99.1%). Prior to completing the LCA, we excluded participants who reported that they had not experienced a romantic relationship between the ages of 13 and 17 (*n*=51; 16.2%); we further excluded three participants who did not provide data for this question, leaving an effective sample size of 264 participants. The majority of remaining participants were female (*n*=215; 81.7%), ranged in age from 18 to 48 (*M*=22.41, SD=6.01), and were Caucasian (*n*=261; 99.2%). Almost the entire sample (*n*=248; 95.0%) reported their sexual orientation as heterosexual. Just less than half of the sample reported being currently single (*n*=139; 41.8%), whereas five (1.9%) reported being widowed, separated, or divorced, with the remaining participants currently in a relationship. For information relating to the endorsement of victimisation subscales, please see Table 1, which details endorsement rates across PA, PAS, injury, and SC subscales across the three exposure periods—parental, adolescence, and adulthood. Based on an assessment of the scores of the psychological correlates within the current study, depression scores ranged from 0 to 27 (*M*=6.86; SD=5.76), PTSD scores ranged from 0 to 53 (*M*=9.84; SD=12.56), alcohol use scores ranged from 0 to 18 (*M*=4.45; SD=3.10), and anger scores ranged from 7 to 39 (*M*=12.75; SD=5.30).

**Table 1:** Victimisation subscale endorsements across the life-course (*N*=264)

	Parents’ relationships, *n* (%)	Adolescence (13–17), *n* (%)	Adulthood (18 +), *n* (%)
Psychological aggression	205 (78.2)	183 (75.6)	190 (79.2)
Physical assault	73 (29.8)	58 (23.8)	73 (30.5)
Injury	15 (5.7)	12 (4.9)	18 (7.5)
Sexual coercion	–	40 (16.4)	64 (26.4)

Sexual coercion within parental relationships was not assessed in the current study. Values are representative of those endorsing one or more experiences in the relevant subscale domain. Categories are not mutually exclusive. %=percent of those who responded.

### Baseline latent class model

**Fig. 1:**
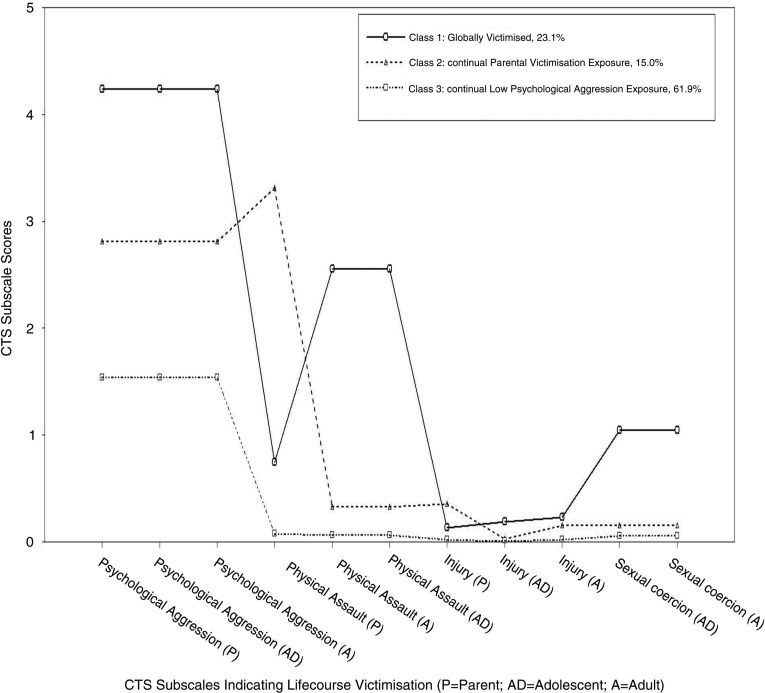
Latent profile plot of CTS subscales across exposure to inter-parental, adolescent, and adult victimisation.

Models were estimated that included 2, 3, 4, and 5 latent profiles. Notably, the loglikelihood was not replicated in the 5-class solution indicating the extraction of too many classes (Nylund, Asparouhov, & Muthén, 2007). The resultant fit indices for the 2–4 class solutions are shown in Table 2. In the current study, values for the AIC, BIC, SSABIC fit indices were lowest for the 4-class solution and the entropy value was highest for the 3-class solution. The BSLRT was not significant for the 4-class solution suggesting that the 3-class solution was more optimal. Given these discrepancies, the differences were calculated between fit statistic values for each solution. Doing so highlighted that differences in fit statistics were minimal when moving from the 3- to the 4-class solution; suggesting again that an additional class added little to the model. This procedure has been suggested by DiStefano and Kamphaus (2006). The optimal model was therefore deemed to be the 3-class solution. The resultant profile plot is shown in Fig. 1. Class 1 comprised 23.1% of the sample, class 2 comprised 15.0% of the sample, and class 3 comprised 61.9% of the sample. Discrimination between the latent classes was good given that the average latent class probabilities for most likely latent class membership was high (class 1=0.96, class 2=0.93, class 3=0.94).

**Table 2:** Fit indices for a two-class, three-class, four-class, and five-class solution using latent class analysis for the 11 CTS subscales

Model	AIC	BIC	SSABIC	Entropy	Lo-Mendell–Rubin adjusted LRT (*p*)
2 class	5701.310	5783.469	5710.548	0.888	781.170 (0.0002)
3 class	5504.658	5629.684	5518.717	0.892	217.400 (0.0015)
4 class	5434.549	5602.441	5453.428	0.877	92.722 (0.6257)
5 class	–	–	–	–	–

AIC=Aikaike Information Criteria; BIC=Bayesian Information Criteria; SSABIC=sample size adjusted BIC.

Class 1 was characterised by high incident rate ratios (IRR) of exposure to PA in their parents’ relationship, and their own adolescent and adult intimate relationships. Relative to the other two classes, the IRRs of exposure to PAS in their parents’ relationships was moderate, despite having the highest IRRs of exposure to PAS in their own adolescent and adult intimate relationships. This pattern continued through the injury subscales. With regard to SC, this class reported the highest IRRs in adolescence and adulthood. Given continued exposure across all subscales pertaining to parent, adolescent, and adult experiences, this class was termed the globally victimised (GV) class.

Class 2 was characterised by moderate IRRs of exposure to PA in their parents’ relationships and their own adolescent and adult intimate relationships, relative to other classes. However, the IRRs relating to PAS in their parents’ relationships was the highest of all groups. The IRRs of exposure to PAS in their own adolescent and adult intimate relationships was considerably lower than in those of class 1 but higher than in those of class 3. This pattern again continued through the injury subscales. With regard to SC, this class reported moderate IRRs in both adolescence and adulthood. Given continued exposure pertaining to victimisation predominately in the parent's relationships, this class was termed the continual parental violence exposure (CPVE) class.

Class 3 was characterised by the lowest IRRs of PA. However, exposure to PA was continual across their parents’ relationship, and their own adolescent and adult intimate relationships. These individuals reported the lowest IRRs across PAS, injury, and SC subscales in their parents’ relationship, and in their own adolescent and adult intimate relationships. Given continued exposure to the PA subscales, this class was termed the continual low psychological aggression (CLPA) class.

### Latent profile psychological correlates

One-way ANOVAs were used to identify differences between the three classes identified above in PTSD scores, depression scores, alcohol use, and anger expression levels (see Table 3 for the descriptive and statistical data related to these tests). All four one-way ANOVAs were significant. Post-hoc comparisons using Tukey HSD revealed class 1 (GV) scores were significantly higher than class 3 (CLPA) scores across all four dependent variables, for example, PTSD (*p*=0.003), depression (*p*=0.008), alcohol use (*p*=0.02), and anger expression (*p*=0.003). Furthermore, class 2 (CPVE) scores were significantly higher than class 3 scores (CLPA) in relation to PTSD (*p*=0.047) and depression (*p*=0.02). All effect sizes were small. No other significant differences were found.

**Table 3:** Results of univariate ANOVAs using class membership as the independent variable and a number of severity scores on psychological correlates as dependent variables

	Class 1: global victimisation (GV)		Class 2: parental victimisation (CPVE)		Class 3: psychological victimisation (CLPA)			
			
Variable	*M*	SD	*M*	SD	*M*	SD	*F*	Partial, *η* ^2^
PTSD (PSS-SR-5)	14.10 (*n*=50)	13.50	13.37 (*n*=27)	12.62	7.14 (*n=*114)	11.46	6.99*	0.07
Depression (PHQ-9)	8.38 (*n=*63)	6.94	8.61 (*n*=36)	6.50	5.83 (*n*=155)	4.76	6.63*	0.05
Alcohol use (AUDIT)	5.62 (*n*=61)	4.14	4.16 (*n=*31)	4.10	4.01 (*n*=142)	3.81	3.70*	0.03
Anger (DARS-7)	14.37 (*n*=62)	5.91	13.97 (*n*=36)	5.48	11.81 (*n*=154)	4.77	6.61*	0.05

* *p*<0.05.

Classes differ in size due to variability of missing data across the different measures.

## Discussion

The first aim was to use LPA to ascertain if underlying typologies of polyvictimisation over the life-course were present in a sample of Northern Irish students. The second aim was to identify if there were mean differences in scores between latent groupings based on a number of known psychological correlates of IPV—PTSD, depression, anger expression, and alcohol use. Thus, the current study examined the heterogeneity of responses to 11 indicators of IPV—PA, PAS, and injury at three points in the life-course (parental, adolescence, adulthood) and SC at two points in the life-course (adolescence and adulthood).

The LPA uncovered three homogeneous groups that differed in the strength of the IRRs and in patterns of endorsement across classes (i.e., not all IRR ratios increased or decreased uniformly in indicators across the various classes). The GV class consisted of those who reported the highest IRRs across all indicators with the exception of parental PAS and parental injury. The CPVE class was characterised by two indicators— parental PAS and parental injury—that spiked in this class compared to the GV and CLPA classes. Also, individuals in the CPVE class reported moderate (relative to other classes) IRRs for parental PA. The CLPA class, which was the largest proportion of the sample, was evidenced by the endorsement of the indicators of PA across all three CTS measures. These indicators were the lowest IRRs of PA relative to the other classes, but they were still notable in their strength of endorsement. The remaining indicators were endorsed at an extremely low or negligible rate. This analysis has demonstrated that there are a proportion of individuals who experience victimisation across various points in the life-course but also across multiple IPV domains, providing evidence for the existence of life-course polyvictims in IPV. This concurs with the recent research regarding polyvictimisation in studies assessing children and young adults (Cavanaugh et al., 2013; Ford et al., 2013; Nooner et al., 2010). Therefore, these findings contribute to our current understanding of IPV by emphasising the importance of examining IPV in the context of multiple forms of violence and extending our knowledge regarding polyvictimisation.

In support of the ITV hypothesis, the current LPA demonstrates that although three latent classes were uncovered, each latent class indicates continuity of violence within the class. The GV class reported the highest IRRs of endorsement across 9 out of 11 indicators. A similar trend was also apparent for the CPVE class in that 9 of 11 indicators were in the moderate range, relative to the two alternative classes. With regard to the CLPA class, all 11 indicators evidenced the lowest IRRs of endorsement. It is unsurprising that each of our latent classes are evidenced by a certain degree of PA, as previous reports have shown that PA is so pervasive within romantic relationships that it could almost be regarded as a normative component (Welsh & Shulman, 2008). Furthermore, this finding corresponds with the Northern Ireland Crime Study reports from 2008 to 2011 (Freel, 2013) that indicate that the most prevalent form of IPV is indeed a form of PA. It is clearly apparent that the level of PA is mirrored across all points in the life-course, suggesting that the level of PA that children are exposed to within their parents’ relationship very closely mirrors the level of PA that they will experience in their own later romantic relationships. Thus, in-line with SLT (Bandura, 1973, 1977), it is reasonable to suggest that when children witness psychologically aggressive interactions between their parents they learn that these interactions are normal and acceptable. Of particular note, the empirical literature has reported that the ITV hypothesis may be more relevant as it pertains to females (Temple et al., 2013). Given that the sample is predominately female, this may partly account for the strong linkage between exposures to inter-parental PA as children and the victimisation from PA as both adolescents and adults (Stith et al., 2000).

Continuity in victimisation was also apparent in the indicators of PAS, injury, and SC; however, this was more apparent when looking at continuity in IRRs for endorsements of events in adolescence and adulthood for both the GV class and the CPVE class. It was less apparent in the CLPA class given that all indicators in this class were endorsed at an extremely low rate, with the exception of the PA indicators discussed above. On one hand this is counter to the ITV hypothesis as it would appear that exposure in childhood does not dictate victimisation in later romantic relationships, but confirms previous research that IPV victimisation in adolescence predicts IPV victimisation in adulthood (e.g., Smith et al., 2003). Furthermore, counter to the ITV hypothesis, the parental PAS and parental injury indicators are elevated in the CPVE class, who report lower levels of PAS, injury, and SC in their own adolescent and adult romantic relationships. Also, the reverse is apparent for the GV class who report less severe IRRs for the parental PAS and parental injury indicators, but report the most elevated IRRs for the PAS, injury, and SC in their own adolescent and adult romantic relationships. This suggests that witnessing severe inter-parental conflict reduces the likelihood of an individual becoming a victim of direct IPV in adolescence and adulthood across multiple IPV domains. SLT (Bandura, 1973, 1977) offers an explanation by proposing that individuals model their behaviour on others not only via the direct observation of the behaviour but also based on their perception of the consequences of such behaviour (e.g., behaviour with positive consequences is more likely to be modelled). Therefore, as witnessing PAS and injury as a result of inter-parental conflict may be associated with negative outcomes, it is likely to be perceived negatively by the observing child. This ultimately reduces the likelihood that the child will model these behaviours in their later romantic relationships (Olsen, Parra, & Bennett, 2010). Moreover, severe negative consequences of inter-parental conflict, such as physical injury, may result in less accepting attitudes of the use of violence in intimate relationships (Malik, Sorenson, & Aneshensel, 1997). In turn, less accepting attitudes of violence reduces the likelihood of an individual becoming involved in violence.

Notably, the degree of victimisation across domains appears to be relatively consistent in that those who report high PA additionally report high PAS, injury, and SC as evidenced by the GV class, but also at moderate levels within the CPVE class. This is particularly evident when looking at polyvictimisation in adolescence and adulthood but not as evident in relation to exposure to parental victimisation. Indeed, looking at just parental indicators in the LPA plot (see Fig. 1), polyvictimisation still occurs, but the indicators across classes do not uniformly increase or decrease. Indeed, PAS and injury exposure dip in those exposed to a high level of inter-parental PA. One explanation could be that parents hide the PASs and injury from children. For example, if children are in adjoining rooms they will be able to “hear” the altercation but won't be able to “see” the PAS (Abrahams, 1994; Hughes, 1992). Likewise, the parents may hide the injury from children or attribute blame for the injury to another non-IPV event; indeed, it has been suggested that injury as a result of IPV may not be disclosed until years after the occurrence of the event (Hamby, Finkelhor, Turner, & Ormrod, 2011). It is important, however, to note that the majority of exposures to inter-parental conflict are regarded as direct/eye witness exposures (Fantuzzo & Mohr, 1999; Holden, 2003).

The GV class, thus the polyvictims, reported a greater degree of psychiatric morbidity, alcohol misuse, and issues with anger expression compared to the CLPA class. This is supported by recent research by Ford et al. (2013) and Cavanaugh et al. (2013). In the current study, there is some evidence that we may have two polyvictims groups that differ in their parental exposure experiences and their severity of exposure to IPV (see Fig. 1). This may explain in part why none of the psychological correlates were able to differentiate between the GV and CPVE groups. Indeed, both depression and PTSD scores were significantly different in the “two” polyvictims groups compared to the CLPA group but not compared to each other.

In the current study, alcohol misuse and anger expression were significantly higher in the GV group compared to the CLPA group only. This finding may be related to the degree of severity of exposure within the GV group in that anger expression and alcohol use is associated with polyvictims who experience the greatest severity of victimisation. Indeed, alcohol has often been associated with polyvictimisation and with IPV victimisation more generally (Cavanaugh et al., 2013; Parks & Fals-Stewart, 2004; Parks, Hsieh, Bradizza, & Romosz, 2008). It is believed that alcohol use increases risk for IPV given alcohol significantly reduces the victim's ability to resist actions committed by perpetrators (Mohler-Kuo, Dowdall, Koss, & Wechsler, 2004).

In relation to anger expression, much of the research interest has focused on anger expression in perpetrators of IPV (see Norlander & Eckhardt, 2005). However, we would also argue that anger expression has a role in victimisation. Indeed, drawing from attachment theory, Dutton and Sonkin, (2003) suggested that individuals, who have an insecure attachment style, as is seen in many IPV victims, may have issues with the regulation of emotions (i.e., anger expression). In addition, the anger that is generated in insecurely attached individuals is thought to be as a function of the loss of a relationship. Typically, victims of IPV feel that they have “lost” a loving relationship when IPV occurs. Thus, it is plausible that this generates anger (Dutton & Sonkin, 2003). In turn, this may explain the significantly increased mean anger expression scores in the GV group compared to the CPVE group identified herein.

### Limitations

The current study is not without limitations. Participants were a convenience sample of university students and it is possible that individuals who are experiencing or have experienced recent IPV may not be currently attending university. Moreover, individuals who are experiencing significant levels of psychiatric morbidity or issues with alcohol misuse may also be absent from university. Self-report measures were used throughout, which give individuals an opportunity to both under- and over-report their experiences. However, self-reporting has been previously documented as being reliable, particularly in relation to self-reports of psychiatric morbidities (Coffey, Dansky, Falsetti, Saladin, & Brady, 1998; Harrington & Newman, 2007). In addition, it is important to note that we had a preponderance of females in the current sample which has the potential to bias the results.

In the LPA, we used count variables as indicators of IPV across different domains. The original data comprised a series of items within a particular domain, and thus it is possible that different items within a particular domain may be regarded as more, or less severe, than other items in the same domain. Adding items to create count variables does not reflect differences in the individual items severity. In a similar vein, we did not assess the frequency with which individual items occurred. It is possible that certain individuals may appear to score low on a given IPV domain based on a low count value. However, this may not necessarily be a true reflection for everyone given that the count variables do not reflect multiple occurrences of single items. On the contrary, given that we did not query frequency or intensity of individual events, creating count scores may have been a better reflection of the true extent of IPV, given that previous reports state that single item indicators can underestimate the extent of events (Bolen & Scannapieco, 1999).

Furthermore, we asked individuals to retrospectively recall occurrences of IPV, for example, between parents; therefore, the length of time from the occurrence of the events and the recall of the event may have impacted upon accuracy. However, the current study was interested in the general recall of certain IPV domains rather than specific details as is evident in our creation of count variables for use in the LPA. Moreover, as we surveyed university students, it is highly likely that a large proportion of these students only very recently left their childhood/parental homes, and so recall of inter-parental conflict may be reasonably recent as to not interfere with the reliability of memories of such events.

## Conclusion

Overall, it is apparent that a proportion of individuals experience both inter-generational transmission and polyvictimisation of IPV events, thus providing evidence for IPV life-course polyvictims. These individuals are shown to experience increased levels of psychiatric morbidity and issues with alcohol misuse and anger expression. This is in line with previous research that supports the ITV hypothesis of violence and the co-occurrence of victimisation events across multiple victimisation domains (Cavanaugh et al., 2013; Ford et al., 2013; Parks & Fals-Stewart, 2004; Parks et al., 2008). It is, however, important to note that these conclusions are based on a convenience sample of university students who volunteered to participate. A number of theoretical positions such as SLT and attachment theory (Bandura, 1973; Bowlby, 1979) provide some explanation of the underlying mechanisms that confer risk of victimisation; however, there is significant room for development in this area. It is important to note that IPV research needs to address several methodological limitations. For example, studies need to standardise the way in which IPV events are both defined and measured as well as understanding the importance of not solely focusing on one form of violence. By adopting these approaches, we can develop a fuller and more generalisable understanding of IPV.
